# Relics of Jenner

**Published:** 1923-03

**Authors:** 


### SOME JENNER RELICS.

### A WIGMORE STREET EXHIBITION.

THE centenary of the death of Dr. Edward Jenner is being commemorated at the Wellcome Historical Medical Museum in Wigmore Street by an interesting exhibition of articles associated with him and his discovery of vaccine inoculation. A number of objects connected with Dr. Jenner and his work have a permanent home in the little Wigmore Street Museum, but to mark the centenary many other relics and documents have been got together and will remain on view during the next few months.

### "Mercenary Spreaders of Death."

The campaign of vituperation which Jenner had to face in the early days of his experiments is recalled by a collection of scurrilous prints. Cruikshank was responsible for a peculiarly grotesque production in 1812, entitled "The Cow Pox Tragedy." This work of art is "dedicated to the Associated Jennerian Cow Poxers of Gloster," and depicts the burial of "Vaccina, who died on April the first." Above the funeral procession, drawn in characteristic Cruikshank style, are rays of light symbolising the triumph of "commonsense, candid investigation, reason, religion and truth" over vaccination. In another caricature the advocates of the new method of small pox prevention are described as "mercenary and merciless spreaders of death and devastation," and the picture shows them being "driven out of society."

### Skill and Perseverance.

It is more agreeable to turn to evidences of a more tolerant and receptive attitude. One may observe, for example, the diplomas presented to Jenner by the University of Cambridge, Massachusetts, in 1803, by the Royal Medical Society of Edinburgh and by the Physical Society of Guy's Hospital in 1802. There is, too, the finely wrought gold and enamelled box given to him in 1803 by the Corporation of London "as a token of their sense of his skill and perseverance in the discovery of and bringing into general use vaccine inoculation." A number of interesting documents are shown, among then the first manuscript of Jenner's "Inquiry into the natural history of a disease known in the Western counties of England as the cowpox," on which he began to work in 1797, the year after his historic experiment of inoculation on the boy Phipps. He begins his paper as follows: "The deviations of man from the state in which he was originally placed by nature seem to have proved to him a prolific source of diseases. From the love of splendour, from the indulgence of luxury and from his fondness for amusement, he has familiarised himself with a great number of animals which may not have been intended for his associates."

### The "Cow with the Crumpled Horn."

There are several portraits of Jenner, and also of his sister and Miss Kingscote, whom he married. The old vicarage at Berkeley, where he was born, is shown in some water-colour drawings. Dr. Jenner inoculating his eldest son (aged 18 months) with swinepox matter is a modern piece of work by Monro Orr, and is certainly more relevant than the "painting of apples from Dr. Jenner's garden," which appear to differ in no important degree from apples grown in anybody else's garden. There is a horn of Jenner's cow "polished by that great benefactor of the human race," says the inscription, "and given by him in 1813 to Sir John W. Fisher," who presented it to the Royal College of Physicians in 1872. In another case is a fragment of hair from the tail of "Blossom," the first cow that furnished vaccine for experiments. Other exhibits include Jenner's walking-stick, vaccine points, lancets, cupping glass and numerous letters. Admission to the museum is free.

